# 3D-printed porous tantalum for acetabular reconstruction in complex primary arthroplasty and revision of hip

**DOI:** 10.3389/fbioe.2025.1557882

**Published:** 2025-05-27

**Authors:** Zonghan Wang, Ze Wang, Lingchuan Gu, Ying Zhang, Tiao Su, Jiangming Luo, Chengjun Huang, Xiaoyuan Gong, Yang Peng, Guangxing Chen

**Affiliations:** ^1^ Center for Joint Surgery, Intelligent Manufacturing and Rehabilitation Engineering Center, The First Affiliated Hospital of Army Medical University, Chongqing, China; ^2^ Chongqing Municipal Science and Technology Bureau Key Laboratory of Precision Medicine in Joint Surgery, Chongqing, China; ^3^ Chongqing Municipal Education Commission Key Laboratory of Joint Biology, Chongqing, China

**Keywords:** total hip arthroplasty, 3D-printed tantalum buttresses, acetabular reconstruction, osseointegration, personalized implants

## Abstract

**Introduction:**

In cases of hip joint damage, such as osteoarthritis (OA), rheumatoid arthritis (RA), avascular necrosis, or hip fractures, total hip arthroplasty (THA) is a critical surgical intervention. For individuals whose hip abnormalities stem from congenital issues, injuries, or previous operations, this procedure can encounter considerable obstacles, including complex bone defects, soft tissue deficiencies, and an increased risk of infections, which may result in poor alignment, joint instability, and higher need for revisions. This study explored the application of personalized, three-dimensional (3D)-printed porous tantalum buttresses designed specifically for acetabular reconstruction. Renowned for its compatibility with human biology, tantalum facilitates superior integration with natural bone.

**Methods:**

The development process started with the generation of meticulous computer-aided design (CAD) models, derived from preoperative imaging techniques such as computed tomography (CT) scans and (magnetic resonance imaging) MRIs, which allowed for the creation of components precisely matching each patient’s unique anatomical structure. The 3D-printed porous tantalum buttresses were made by cutting-edge additive manufacturing methods. The porosity of the tantalum structure promoted the growth of new bone tissue into the implant, improving its stability and durability. During surgeries, the buttress was positioned to reconstruct the acetabulum, laying a solid foundation for the artificial hip joint.

**Results:**

The results of our study showed that all surgeries were successfully completed with no significant vascular or nerve damage. Postoperative evaluations showed that the buttress had excellent biomechanical function and firm fixation, with a large amount of bone ingrowth, improving the fitness and performance of the implant while reducing the possibility of subsequent problems such as loosening or dislocation.

**Discussion:**

This innovative technique has great potential in clinical practice for better outcomes and quality of life for patients with complex hip deformities.

## 1 Introduction

Total hip arthroplasty (THA) for acetabular deficiency has always been challenging, particularly in patients with a history of previous surgeries, joint ankylosis, infection of previous medical history, and extensive bone defects (Patel et al., 2023; Ruff et al., 2024; Patel and Golwala, 2023). These patients often present with substantial and irregular bone defects, severe soft tissue deficiencies, residual dormant bacteria, and repositioning of critical structures such as blood vessels and nerves, which complicates the repair of bone and soft tissue and increases the risk of infection recurrence, neurovascular disease, and other complications. Acetabulum bone defect will affect the stability of acetabulum prosthesis implantation, finally causing the failure of surgery (Ying et al., 2023; Xu et al., 2023; Hettich et al., 2019). However, repairing deformed bone defects after various reasons has consistently posed challenges and controversies. Obtaining allogeneic or autologous bone grafts is inherently difficult due to increased risk of infection; therefore, their application is limited. The use of metal materials has become the primary approach for repairing bone defects with significant progress being made in this area. Nevertheless, current normal titanium alloy and tantalum products often do not conform to the shape of the host bone, leading to difficulties in implantation, excessive loss of host bone, neurovascular injury and poor fixation and osseointegration, ultimately resulting in failure (Chiarlone et al., 2020; Ferraz, 2023; Schierjott et al., 2020).

Acetabular bone defects, often caused by trauma, infection, tumors, osteolysis, or congenital disorders, are a significant challenge for THA reconstruction. Choosing the right materials and preparing the repair components are critical to the success of the procedure. Tantalum is known for its excellent biocompatibility and mechanical properties, making it the best choice for bone replacement and repair (Wen et al., 2021). By fine-tuning the porous structure, tantalum implants with suitable elastic modulus can be fabricated to bridge the gap between cancellous and cortical bone, thereby reducing the risk of stress shielding. The material’s high coefficient of friction enhances the stability of the initial implant, while its trabecular design promotes the growth of new bone tissue. Currently, Zimmer’s vapor deposition process is widely used to fabricate porous tantalum prostheses for bone defects (Mora et al., 2022; Yu et al., 2024; Peng et al., 2024). However, after more than 2 decades of application, there are still many shortcomings, including the difficulty of accurately matching the implant shape to the bone defect, and the possible increase in bone loss and infection during bone modification. The 3D printing technology enables the production of complex shapes, which can provide precise prosthesis customization (Hua et al., 2022; Hua et al., 2021; Ying et al., 2023; Ao et al., 2022). Specifically, the application of computer-aided design (CAD) and finite element analysis (FEA) has played a vital role in hip joint reconstruction, enabling preoperative biomechanical simulation and optimization of implant design. These methods help evaluate stress distribution, improve the mechanical compatibility of implants, and reduce the risk of postoperative failure. For instance, some researchers conducted structural analysis of the articular cartilage of the hip joint using FEA to understand joint load distribution (Karpiński et al., 2016). Similarly, other researchers analyzed the pelvic girdle pre- and post-hip replacement, highlighting key insights into implant stress behaviors (Zubrzycki et al., 2018). Including such modeling in personalized implant design contributes significantly to biomechanical performance and patient safety. The 3D printed porous tantalum prostheses can be made using electron beam as an energy source in a vacuum environment, with the advantages of rapid prototyping and protection against gas oxidation and impurity contamination.

In this study, we managed two cases of complex deformity with a significant acetabular bone defect following hip joint infection and one case of complex deformity with a significant acetabular bone defect following rheumatoid arthritis (RA). The bone defect was repaired using a personalized 3D-printed porous tantalum buttresses, and THA was performed (Jiao et al., 2023; Zhang et al., 2023).

## 2 Materials and methods

### 2.1 Materials

#### 2.1.1 Tantalum powder

**FIGURE 1:**
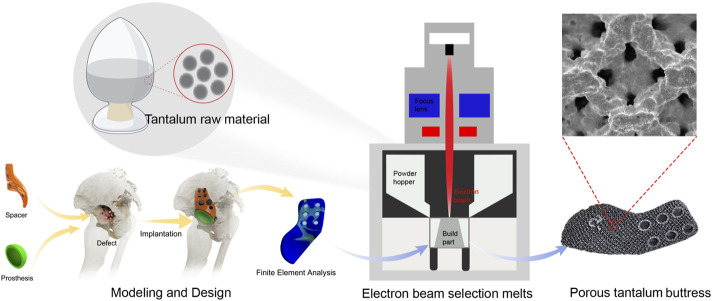
Fabrication process of tantalum metal and buttress.

The 3D-printed porous tantalum buttress was made of Ta1 type I tantalum powder from the Northwest Institute for Nonferrous Metal Research in China (Figure 1). The primary element in its chemical composition is Ta. The impurity elements (mass fractions ≤, %) are as follows. O: 0.030, C: 0.010, N: 0.010, H: 0.0020, Nb: 0.050, Fe: 0.010, Ti: 0.005, W: 0.010, Mo: 0.010, Si: 0.005, Ni: 0.010. The Ta1 type I tantalum powder is spherical and has the following physical properties: particle size ranging from 45 μm to 105 μm, an apparent density ≥9.2 g/cm^3^, a tapped density ≥9.8 g/cm^3^, and a flowability ≤10 s/50 g.

#### 2.1.2 Patients

**FIGURE 2:**
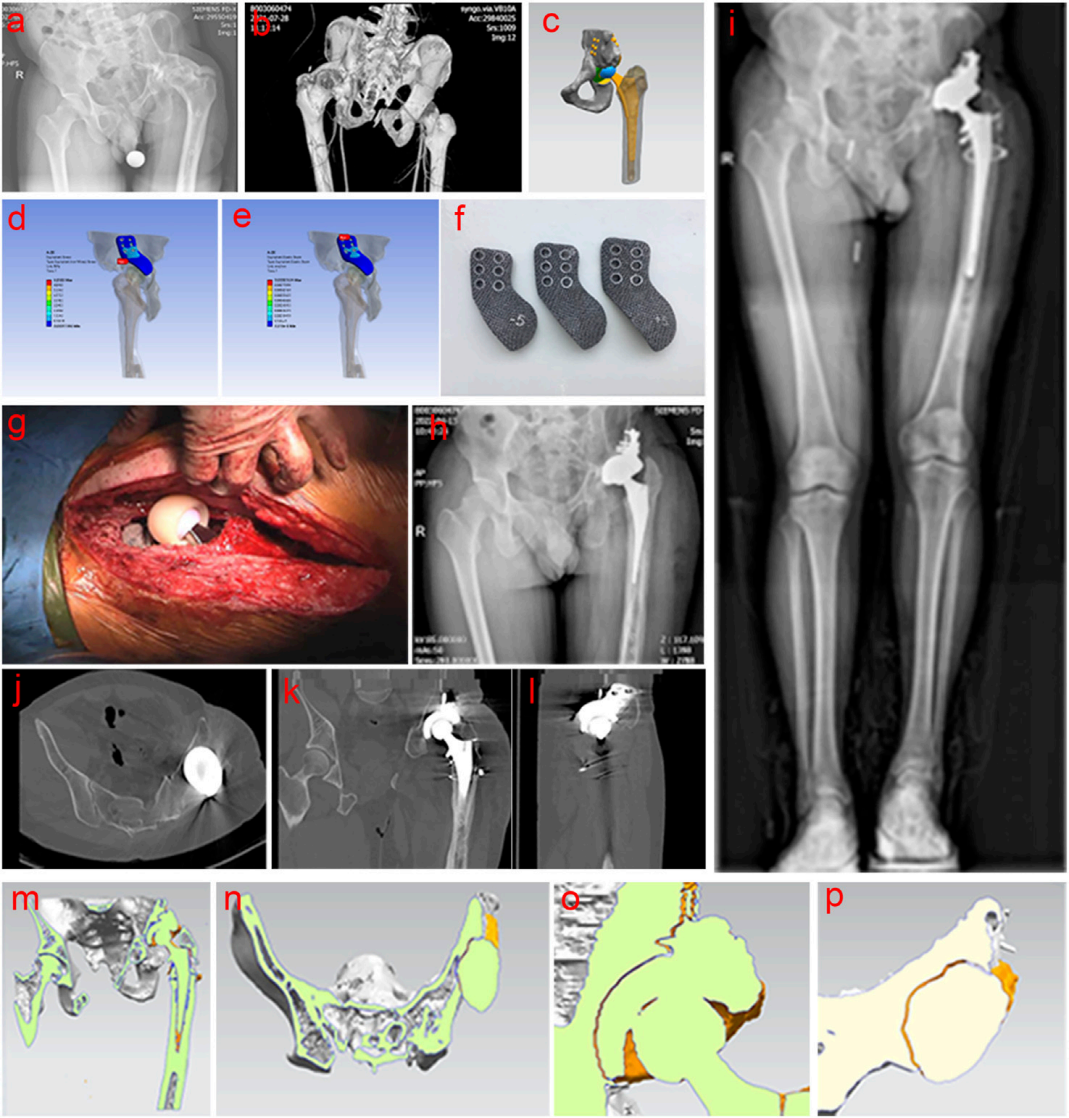
Clinical data of Case 1. **(a,b)** Preoperative examination and evaluation; **(c–f)** Personalized implant design, verification and printing; **(g–l)** Intraoperative operation and postoperative imaging examination; **(m–p)** Comprehensive analysis of bone ingrowth based on postoperative imaging data.

**FIGURE 3:**
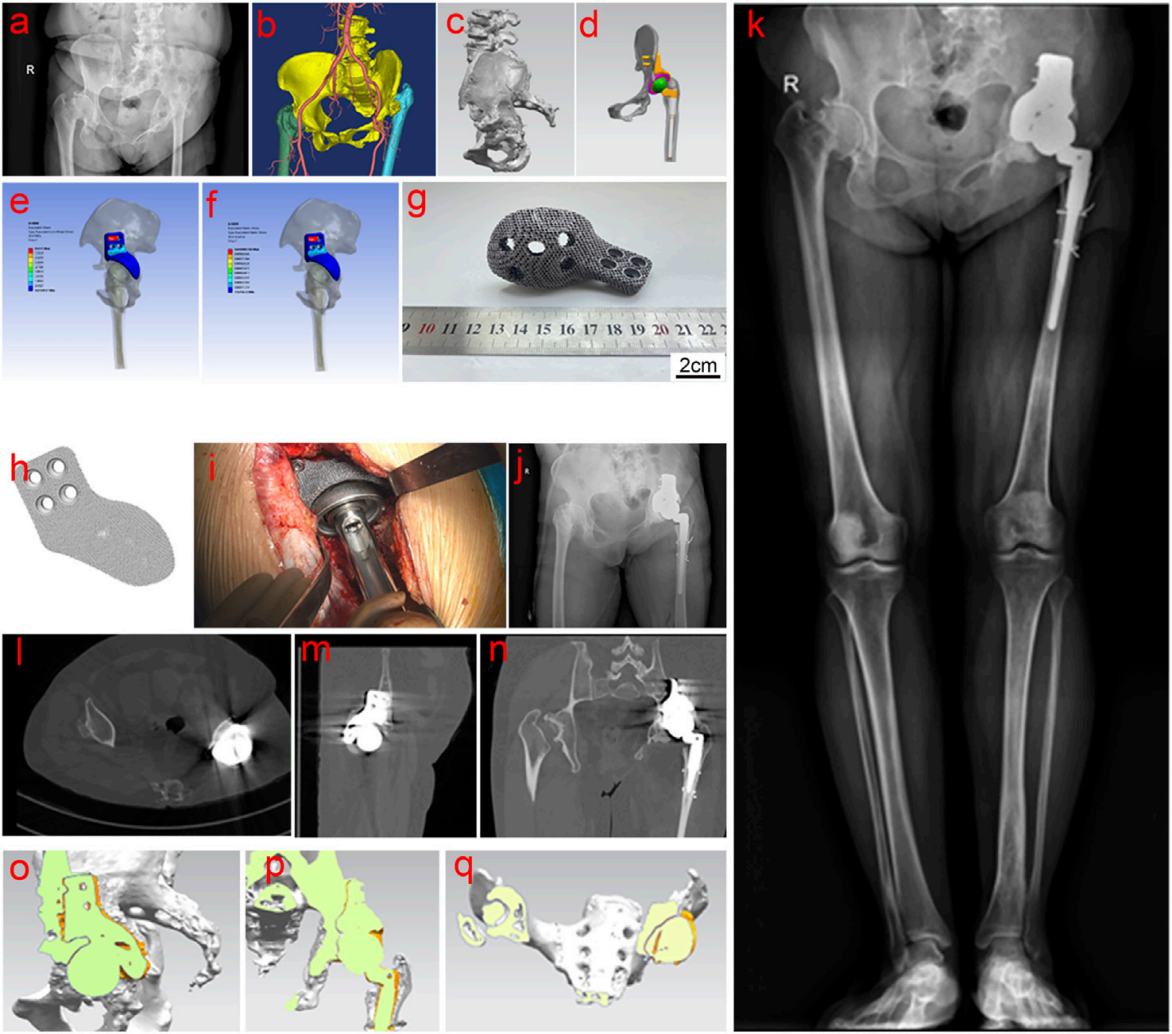
Clinical data of Case 2. **(a–c)** Preoperative examination and evaluation; **(d–h)** Personalized implant design, verification and printing; **(i–n)** Intraoperative operation and postoperative imaging examination; **(o–q)** Comprehensive analysis of bone ingrowth based on postoperative imaging data.

**FIGURE 4 F4:**
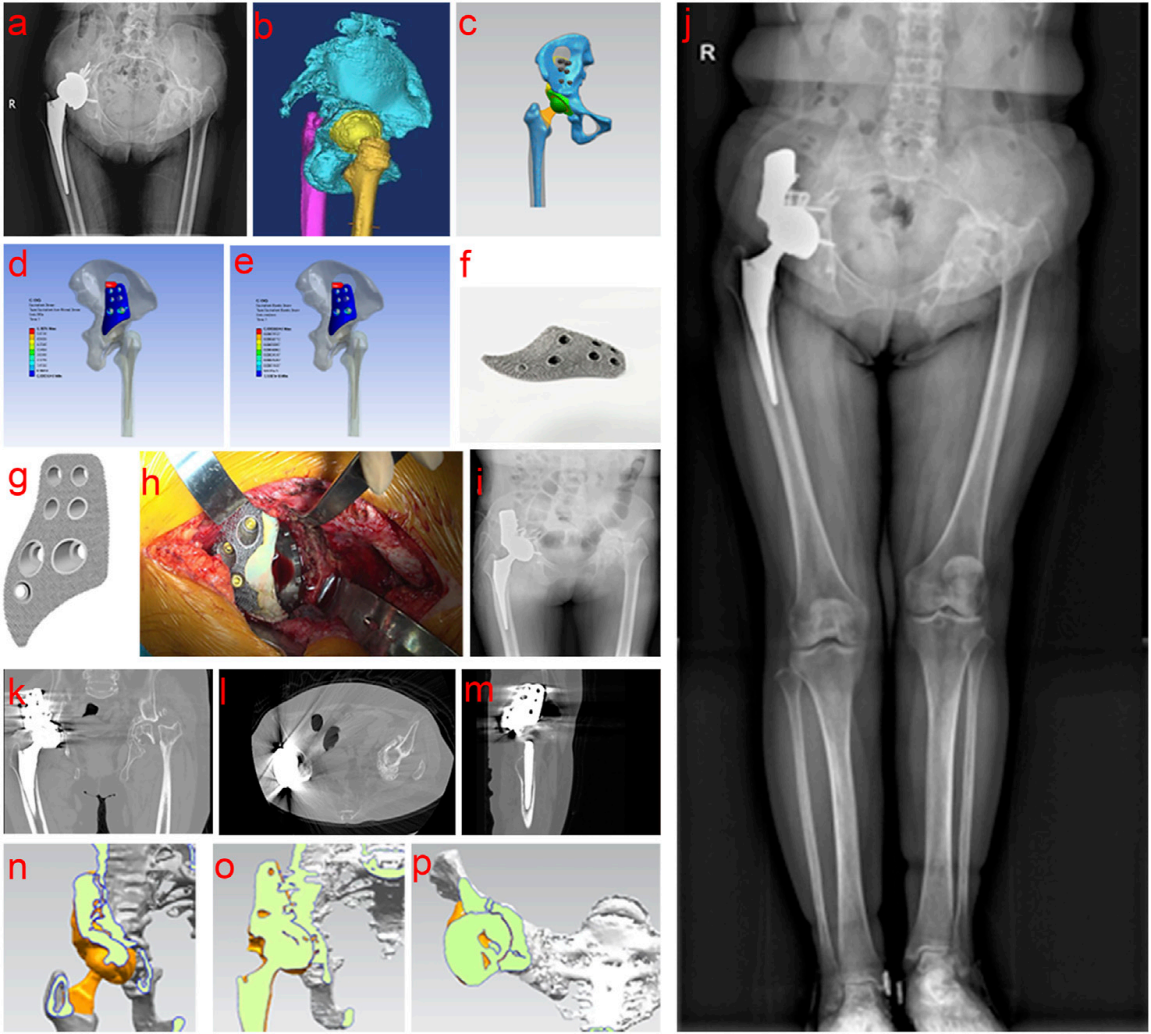
Clinical data of Case 3. **(a,b)** Preoperative examination and evaluation; **(c–g)** Personalized implant design, verification and printing; **(h–m)** Intraoperative operation and postoperative imaging examination; **(n–p)** Comprehensive analysis of bone ingrowth based on postoperative imaging data.

Three patients with acetabulum bone defect who underwent THA using 3D-printed porous tantalum buttress in our Center from July 2021 to March 2024 were selected. Among the three patients, two had sequelae of infection, and one had severe RA, which resulted in acetabular destruction and pelvic discontinuity. Specifically, Case 1 was a 32-year-old male patient with left pelvic fracture and infection caused by a traffic accident, which was cured after more than 10 times of debridement without internal fixation. The patient had left hip ankylosis in flexion and adduction of 15° and 20°of left hip. The course of disease was 11 years (Figures 2a,b). Case 2 was a 55-year-old woman, with left hip infection, postoperative pain, limping for 15 years, worsened over the past 2 years (Figures 3a–c). Case 3 was a 52-year-old female presented with bilateral hip destruction due to RA, with chronic pelvic discontinuity, and acetabular cup loosening 2 months after right THA (Figures 4a,b). All three patients had huge acetabular bone defects, and conventional hip arthroplasty could not maintain the stability of the acetabular prosthesis. All patients complained of hip pain after standing for a long time or walking long distances, limited motor functions such as squatting, running and jumping; all patients had claudication. All patients received conservative treatment for more than 3 months, and their symptoms did not improve, affecting their daily life and work.

This study was approved by the Ethics Committee of our hospital (No. KY201908), and all patients signed the informed consent form.

### 2.2 Workflow of design and fabrication of porous tantalum buttress

#### 2.2.1 Imaging and CAD model reconstruction

**FIGURE 5:**
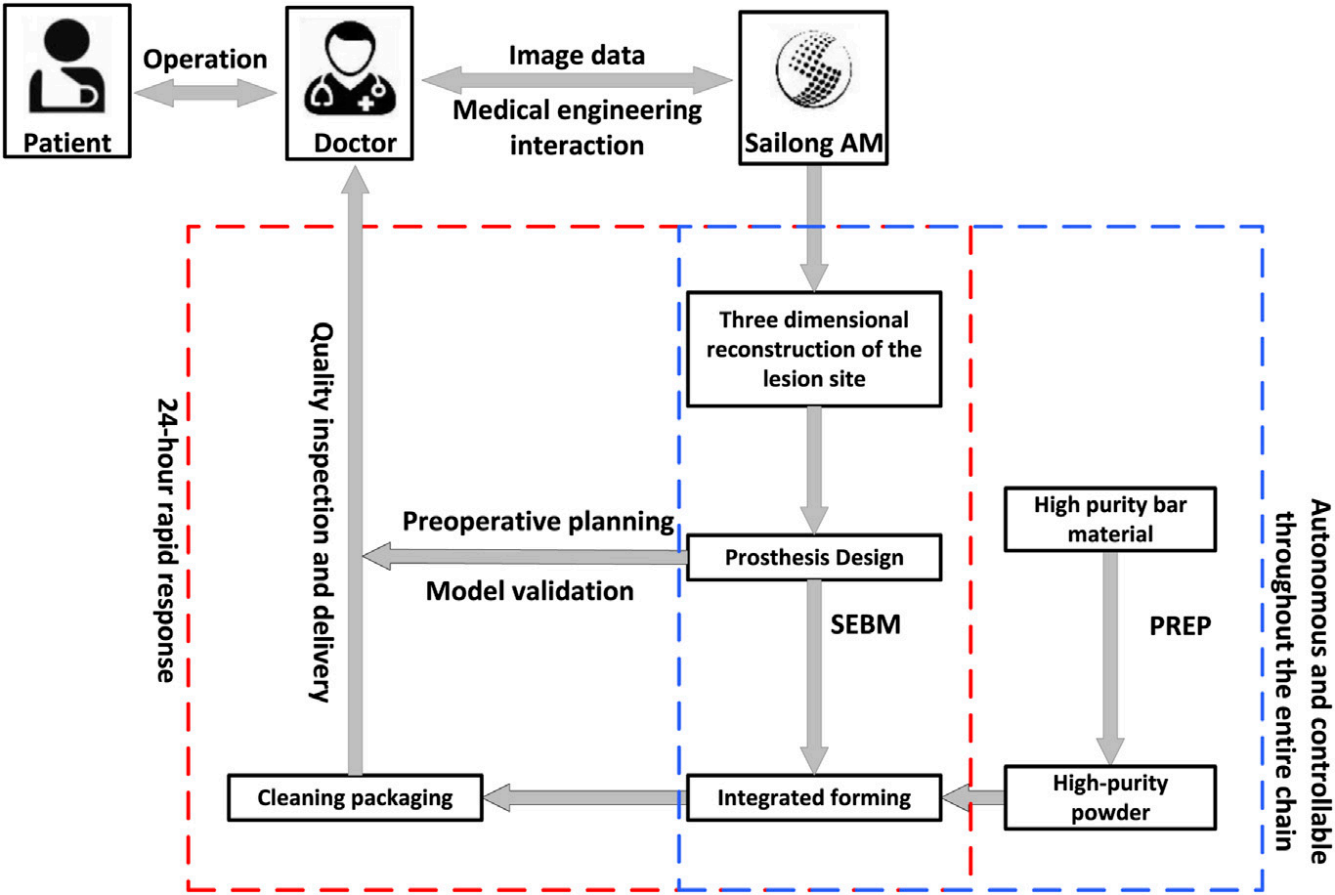
Workflow diagram for personalized prosthesis design and manufacturing using medical engineering and 3D printing.

Computed tomography angiography (CTA) of lower limbs were scanned using the SOMATOM Definition computed tomography (CT) scanning equipment (SIEMENS, Germany), with slice thickness and spacing set at 1.0 mm. The scanning range included the entire pelvis and the proximal 15 cm of both femurs, with a resolution of 512 × 512 pixels. While ordinary CT scans cannot display blood vessels, the application of CTA can visualize both blood vessels and bone positions, allowing designers to avoid blood vessels during the design process. The two-dimensional (2D) CT images were converted into digital image correlation method (DICM) format using INFINITT software (Infinitt, China). Patient CT data was imported into Mimics Research 19.0 software (Materialise, Belgium). Threshold-based segmentation and dynamic segmentation were performed to extract masks for the pelvis, sacrum, and the proximal ends of both femurs, as well as blood vessels, which were converted into 3D models. These models were then exported into STL format and reverse-engineered using Geomagic Studio software (Geomagic, United States) to generate STP format data. Deviation analysis was performed on the STP data before and after reverse engineering to provide foundational digital models and design references for the structural design of components for patients with deformities following hip infection (Figure 5).

#### 2.2.2 Prosthetic material properties & design principles of buttress

Data related to material properties were obtained from the Chinese studies by Gu and Wang (2024) and Fu et al. (2018). Based on these data, the stress and strain distributions of the tantalum buttress were calculated accordingly (Table 1).

**TABLE 1:** Prosthetic material properties.

Materials	Young modulus	Poisson’s ratio	Items
Cortical bone	5,600	0.30	
Cancellous bone	500	0.30	
Tantalum metal	5,963	0.31	Tantalum metal cover
Polyethylene	800	0.45	Liner
Titanium alloy	110,000	0.30	Screw, Femoral components
Porcelain	350,000	0.22	Head of femoral acetabular cup
Vitallium	210,000	0.30	

The STP file was imported into the UG software (Unigraphics NX, United States), establishing a coordinate system in a normal standing position, and adjusting the 3D spatial position of the pelvis to ensure it was fully placed in a neutral position. The design of the porous tantalum buttress followed these principles: (1) Drawing lessons from previous patient’s case of personalized metal implant, the irregular design helps avoid blood vessels; (2) Simulate the placement of the implant based on clinical needs, and design the buttress after determining its position; (3) Only retain osteophytes that are in contact with bone support, and remove the rest; (4)The wing of the buttress is designed to be fixed at the middle 1/3 position of the ilium; (5) The posterior side of the buttress can be partially reduced, not requiring full coverage; an additional screw hole should be added in the posteriorsuperior region to facilitate screw fixation for the acetabular cup.

The entire model structure included cortical bone, cancellous bone, tantalum buttress, screws, acetabular cup, liner, femoral head, and femoral stem. Prosthetic data of the first patient: femoral components (M/L Taper No. 12), acetabular cup 60 mm, femoral head 32 mm (Figure 2c). Prosthetic data of the second patient: femoral components (Sivash-Range of Motion, S-ROM 14*8*130 oversleeve 14B), acetabular cup 48mm, femoral head 32 mm (Figure 3d). Prosthetic data of the third patient: femoral components (M/L Taper No. 6), acetabular cup 56 mm, femoral head 32–3.5 mm (Figure 4c). The elastic modulus of human cancellous bone is 0.1–1.5 GPa, while the elastic modulus of cortical bone is 12–18 GPa. In order to avoid stress shielding, the prosthesis for acetabular bone defect should be designed with a porous structure (pore diameter 250–550 μm, filament diameter 250–450 μm), and the elastic modulus should be reduced to about 3 GPa, which is between cortical bone and cancellous bone. The above design takes into account the optimization of mechanics while meeting the clinical needs.

#### 2.2.3 Finite element analysis

Firstly, the physical problems to be analyzed were clarified, including boundary conditions, loads, and material properties. A geometric model of the object to be analyzed was created using modeling tools built into CAD software or finite element analysis (FEA) software. The geometric model was divided into a finite number of small elements (mesh), and the appropriate element type (such as triangle, quadrangle, cube, etc.) and mesh density were selected. The quality of the mesh had an important influence on the analysis results. Second, material properties such as elastic modulus, Poisson’s ratio, density, etc. were defined for each element. According to the problem definition, the corresponding boundary conditions (such as fixation, sliding, etc.) and external loads (such as force, pressure, temperature, etc.) were applied. Finally, FEA software was used for numerical solution to calculate the displacement, stress, strain and other results of each node. The solution results were visualized and analyzed to generate stress, displacement, and other distribution maps to evaluate the safety and performance of the structure (Figures 2d,e, 3e,f, 4d,e).

#### 2.2.4 Verification of the 3D-printed porous tantalum buttress

Life-size models of the acetabulum, acetabular buttress, and femoral head were created using STL file data and polylactic acid (PLA) to ensure an accurate fit and coverage. These components were assembled for surgical simulations to check the alignment between the parts and the femoral head coverage by the acetabulum. If the initial validation did not meet the criteria, adjustment and re-simulation were conducted until the best fit was achieved. The target for ideal acetabular coverage was set at 80%. The position and shape of the acetabular buttress has been carefully adjusted to avoid interfering with blood vessels and nerves, providing sufficient space for screw fixation, enhancing surgical exposure, and ensuring strong prosthetic coverage and mechanical support.

#### 2.2.5 Additive manufacturing process

The porous tantalum buttress was fabricated using a Sailong Y150 powder bed electron beam system (Sailong China), and the operating parameters were detailed in Table 2. The baseplate was preheated in a vacuum. A layer of tantalum powder was evenly spread on the baseplate, which was subsequently heated to over 600°C using an electron beam, facilitating sintering between the powder particles and stabilizing the powder bed. With a maximum power output of 3,000 W, the electron beam provided enough energy to melt tantalum, which had a high melting point of 2,996°C, for precise molding according to design specifications.

**TABLE 2:** Parameters of electron beam selective melting additive manufacturing equipment.

Items	Equipment parameters
External dimensions	2,000 mm × 1,200 mm × 2,150 mm
Forming warehouse size	200 mm × 200 mm × 300 mm
Maximum forming size	150 mm × 150 mm × 200 mm
Powder laying thickness	35–200 μm
Electron beam spot diameter	≤0.2 mm
Dimensional accuracy of formed parts	±0.3 mm
Equipment ultimate vacuum	<9.5 × 10^−4^ Pa

The slicing software allowed the electron beam to selectively melt the tantalum powder following the design data of the buttress. The unmelted powder descended through the build chamber and a new layer of powder was then deposited, repeating this process layer by layer until a porous structure was fully formed.

After printing, the buttress was gradually cooled to room temperature in a vacuum, and then taken from the chamber. Compressed air and metal powder particle impact were used to remove all excess unmelted powder from the surface and internal cavities of the buttress. The buttress was then ultrasonically cleaned in pure water to remove residual material, and then dried, completing the manufacturing process (as shown in Figures 2f, 3g,h, 4f,g). Standardized procedures were followed throughout production process (Figure 1).

### 2.3 Surgical techniques

#### 2.3.1 Case 1

The operation was performed in two stages, and the interval between surgeries was 6 months. In the first stage, according to the shape of the 3D-printed buttress, the bone of the upper edge of the acetabulum was polished and shaped with a grinding drill, and the position was satisfied and temporarily fixed with Kirschner wire. Fluoroscopy confirmed that the articular surface of the buttress matched the radian of the reeled acetabulum, and the femoral head was covered well. Bone cement spacer with appropriate size was prepared according to the morphology and implanted into the joint. The second stage surgery was for taking out the bone cement spacer and artificial total hip revision procedures (Figures 2g,h).

#### 2.3.2 Case 2

During the procedure, the scar tissue was removed and soft tissue release was performed around the hip joint. To ensure optimal exposure and alignment, additional bone cutting was performed in the greater trochanteric region of the checkpoint. Under C-arm fluoroscopy, the position of the acetabular cup was determined using a positioning needle. The acetabulum was gradually enlarged to 52 mm and the grinding drill was shaped along the upper edge of the acetabular bone according to the 3D-printed buttress. Fluoroscopy confirmed that the buttress’ articular surface was perfectly aligned with the curvature of the acetabulum, ensuring that the reamer head was adequately covered. Four screws were placed to secure the buttress, followed by a 54 mm highly cross-linked polyethylene liner with bone cement and adjustments to the femoral bone marrow cavity. After thorough cleaning of the medullary cavity with a pulse irrigator, a 14*8*130 mm S-ROM femoral stem prosthesis was implanted, followed by a 36 mm trial femoral head. The hip joint was successfully reduced. The test confirmed its stability, and the flexion and extension were satisfactory. To accommodate lower limb extension, the right leg was positioned in a hip and knee flexion position. C-arm fluoroscopy confirmed the correct size and position of the hip prosthesis, and a 36 mm ceramic femoral head was then implanted (Figures 3i,j).

#### 2.3.3 Case 3

A specialized tool was used to remove loose acetabular cup, ceramic liner, and femoral head prosthesis. The acetabulum was trimmed to remove the surrounding scar tissue as well as the synovial tissue within the joint. Examination showed that fibrous connections remained firm at the base of the acetabular pelvic discontinuity, while the bones in the superior part were loose. A 3D-printed porous tantalum buttress was placed over the acetabulum. After precise adjustment, the buttress was fixed with five screws. To promote bone healing, the acetabular surface was carefully grounded using abrasive tools, which might lead to pinpoint bleeding in cancellous bone and exposed areas of bone defects. The acetabular cup was reinforced with an additional five screws, and any gap between the buttress and acetabular cup was filled with bone cement for stability. A new 32 mm polyethylene liner was installed, and gauze was temporarily placed inside the acetabulum to protect the components. The femoral stem remained unchanged and was firmly fixed. A 32 mm + 3.5 mm test femoral head was used to ensure smooth hip reduction, and normal flexion and extension were tested. After testing, a 32 mm + 3.5 mm ceramic femoral head prosthesis was implanted.

### 2.4 Postoperative rehabilitation

Immediately upon recovery from anesthesia, ankle pump exercises and static contractions of the quadriceps for the affected limb were initiated; straight leg raises were conducted on the first postoperative day to strengthen the temoriss and lower limb muscles. Within 6 weeks after surgery, partial weight-bearing activities on the affected limb could be conducted with the aid of crutches. By the 7th week after surgery, the affected limb could bear full weight while walking and strengthening exercises for the hip abductor muscles were intensified.

### 2.5 Follow-ups and efficacy evaluation

#### 2.5.1 Intraoperative data and follow-ups

Surgery duration, amount of intraoperative bleeding, and any complications were recorded. On the first day after surgery, pelvic radiograph was conducted to observe the position of the implanted buttress and its fit with the acetabulum and femoral head. Patients were followed in the outpatient department at 6, 13, and 24 months postoperatively.

#### 2.5.2 Subjective evaluation indicators

Patient-reported outcomes (PROs) were recorded preoperatively and at the final follow-up, including the Non-Arthritic Hip Score (NAHS), Modified Harris Hip Score (mHHS), Hip Outcome Score-Exercise Subscale (HOS-SSS), International Hip Outcome Tool-12 items (iHOT-12), and Visual Analogue Score (VAS) for Pain. At the final follow-up, patient satisfaction was rated on a scale of 0–10, ranging from very dissatisfied (0–2) to very satisfied (9–10). The overall satisfaction rate was calculated as follows: (number of patients reporting satisfactory or very satisfied)/total number of cases × 100%.

#### 2.5.3 Postoperative stability of the porous tantalum buttress

The postoperative stability and integration of the porous tantalum buttress were objectively evaluated through radiological examinations. At the final follow-up, imaging examinations were conducted to assess bone ingrowth into the support and to check for any translucent lines that indicated a possible gap between the support and the ilium.

#### 2.5.4 Definition of clinical success

Clinical success in this study was defined based on a combination of objective and subjective criteria. A patient was considered to have achieved clinical success if the following conditions were met at the final follow-up:

(1) No major postoperative complications such as infection, loosening, or revision surgery;
(2) Radiographic evidence of stable implant fixation and osseointegration without progressive radiolucent lines;
(3) Statistically significant improvement in at least three of the five functional outcome scores (NAHS, mHHS, HOS-SSS, iHOT-12, VAS);
(4) A patient satisfaction score of ≥7 out of 10.

These combined criteria were used to provide a comprehensive assessment of both clinical and functional outcomes.

### 2.6 Statistical analysis

Continuous variables in this study were expressed as mean ± standard deviation (SD). For the comparison of pre-operative and final follow-up scores, a paired *t*-test was used, as the data consisted of repeated measures from the same patients at two time points. The significance level was set at *P* < 0.05. All analyses were performed using IBM SPSS Statistics for Windows, Version 25.0 (IBM Corp., Armonk, NY, United States).

## 3 Results

### 3.1 Perioperative data

All three patients (three hips) underwent successful surgeries without any major vascular or nerve injuries. The surgical time per hip ranged from 2 to 2.5 h, the intraoperative blood loss ranged from 100 to 200 mL. All patients’ surgical incisions healed excellently, and no complications such as infection or thrombosis occurred.

### 3.2 Biomechanical evaluation results

#### 3.2.1 Mechanical analysis and evaluation

(1) Reasonable analysis model building. A reasonable analysis model was built, including the physical design and FEA of various parameter settings.
(2) Uniform mechanical distribution. The mechanical distribution on the contact surface with the iliac crest was relatively even, which aligns with the original design.
(3) Metal fatigue strength design. The stress value was less than 10 MPa, which was significantly below the threshold, indicating that the metal fatigue strength design was reasonable.

#### 3.2.2 Patients’ tantalum metal stress distribution results

The FEA results showed that the stress distribution of tantalum buttress was reasonable, which was in line with the original design intention, and the maximum stress point was the edge of tantalum buttress, the maximum stress range being 6.3816–8.541 MPa, with an average of 7.26 ± 1.13 MPa.

#### 3.2.3 Patients’ tantalum metal strain distribution results

The FEA results showed that the tantalum metal strain distribution was reasonable, which complied with the design requirements. The location of maximum strain was also at the edge of the tantalum buttress, consistent with the stress distribution. The equivalent plastic strain ranged from 0.00087634 to 0.00099732 mm/mm, with an average of 0.00092 ± 0.00007 mm/mm.

### 3.3 Evaluation of postoperative stability of the porous tantalum buttress

After reviewing all patients for at least 3 months postoperatively, radiographs at the final follow-up indicated satisfactory bone ingrowth into the porous tantalum buttress, with no translucency lines observed between the implant and the ilium. Comparing the radiological indicators from the immediate postoperative images with those at the final follow-up, no cases showed displacement or bone resorption (Figures 2i, 3k, 4j).

Three-dimensional reconstructions of the hip joint showed that the buttress and prosthesis were stable, with good bone ingrowth and no evidence of displacement or bone resorption (Figures 2j–p, 3l–q; 4k–p, [Fig F4]).

### 3.4 Results of comprehensive assessment

Patients were followed for 9–24 months, with an average of (15.33 ± 6.74) months. Significant improvements were observed across all outcomes. The NAHS increased from 48.67 ± 1.53 to 71.67 ± 5.51, with a mean improvement of 23.00 points (95% CI: 9.86–36.15; *t* = 7.529; *P* = 0.017). The mHHS rose from 54.33 ± 3.51 to 83.67 ± 2.52, yielding a mean difference of 29.33 points (95% CI: 14.36 to 44.31; *t* = 8.429; *P* = 0.014). Similarly, the HOS-SSS improved from 47.67 ± 8.51 to 80.67 ± 5.03, showing a mean change of 33.00 points (95% CI: 24.04–41.96; *t* = 15.853; *P* = 0.004). The iHOT-12 increased markedly from 34.67 ± 17.16 to 77.00 ± 9.54, representing a mean improvement of 42.33 points (95% CI: 22.10–62.57; *t* = 9.003; *P* = 0.012). In contrast, the VAS for pain significantly decreased from 4.33 ± 0.58 to 0.67 ± 0.58, with a mean reduction of −3.67 points (95% CI: −6.54 to −0.80; *t* = 5.500; *P* = 0.032). At the final follow-up, one patient (33.3%) was classified as “satisfied” with a score of eight and 2 patients (66.7%) was classified as “very satisfied” with a score of 9. The overall satisfaction rate (the percentage of patients who were satisfied or very satisfied) was 100% (Table 3).

**TABLE 3:** Results of comprehensive assessment.

Outcome measurements	Pre-operative	Final follow-up	Mean difference (95% CI)	*t*	*P*-value
NAHS	48.67 ± 1.53	71.67 ± 5.51	23.00 (9.86–36.15)	7.529	0.017
mHHS	54.33 ± 3.51	83.67 ± 2.52	29.33 (14.36–44.31)	8.429	0.014
HOS-SSS	47.67 ± 8.51	80.67 ± 5.03	33.00 (24.04–41.96)	15.853	0.004
iHOT-12	34.67 ± 17.16	77.00 ± 9.54	42.33 (22.10–62.57)	9.003	0.012
VAS	4.33 ± 0.58	0.67 ± 0.58	−3.67 (−6.54 to −0.80)	5.500	0.032

NAHS, Non-Arthritic Hip Score; mHHS, modified Harris Hip Score; HOS-SSS, Hip Outcome Score-Sports Subscale; iHOT-12, International Hip Outcome Tool-12; VAS, visual analog scale.

## 4 Discussion

This study confirms the effectiveness of personalized 3D-printed porous tantalum buttress in addressing the complexity of acetabular reconstruction in patients with extensive bone defects. The surgeries were successful in all patients with no significant vascular or neurological complications. The superior biomechanical properties and postoperative stability of pure tantalum buttress, as well as strong bone ingrowth, highlights the promise of this innovative technique (Kong et al., 2022; Liu et al., 2022; Wang et al., 2020).

Previous studies have shown that patients with large acetabular bone defects often face challenges with standard titanium and tantalum implants, such as poor fitness and inadequate osseointegration (Griseti et al., 2020; Peng et al., 2024). This study demonstrates the biocompatibility and mechanical advantages of tantalum, while showing substantial improvements that 3D printing technology can bring. While traditional methods can lead to excessive bone loss, suboptimal fixation, and neurovascular injury, the precision of patient-specific implants significantly enhance the match of individual anatomy (Mitra, 2021; Carraro and Bagno, 2023; Zhang et al., 2021; Marongiu et al., 2023). Compared with conventional implants, 3D-printed porous tantalum implants have a lower elastic modulus (close to human cancellous bone) in terms of mechanical properties, which can effectively reduce the stress shielding effect. In contrast, traditional titanium alloys are more likely to produce stress concentrations at the implant site due to their higher elastic modulus, increasing the risk of bone resorption. In terms of bone ingrowth, the surface of traditional titanium alloys usually needs to be coated with biological coatings such as hydroxyapatite to promote bone ingrowth, while porous tantalum materials themselves have good osteoinduction properties, and their porous structure can provide a larger attachment surface for bone cells and promote the rapid growth of new bone tissue (He et al., 2024). In terms of clinical efficacy, all three patients in this study achieved early stability and did not experience loosening or bone resorption during the follow-ups. In terms of cost effect, although the price of tantalum powder (8,000 CNY/kg) is higher than that of titanium alloys (400 CNY/kg), its porous structure can reduce or replace the need for bone grafting, thereby indirectly saving surgical costs. Compared with standardized implants, individual designs require additional time for image analysis, 3D modeling, and parametric design. In addition, the application of 3D printing technology will also increase the initial manufacturing cost. However, 3D-printed porous tantalum implants can reduce postoperative complications and reoperation rates while shortening recovery time, potentially saving treatment costs (Zhao et al., 2024).

The technique presented in this study is of clinical significance. The personalized fit of 3D-printed implants improves initial stability and promotes osseointegration, which is critical for long-term success. By reducing the need for allogeneic or autologous bone grafts, this method minimizes the risk of infection associated with the donor site. In addition, customized implants can lead to better functional outcomes and higher patient satisfaction, potentially shorten recovery times and improve overall quality of life (Zhao et al., 2024; Hao et al., 2023). Based on the preliminary results of this study, patients using 3D-printed porous tantalum implants demonstrated good functional recovery during the rehabilitation process. Despite the promising findings, this study has several important limitations. First, the sample size was limited to only three patients, which severely constrains the generalizability of the results. The absence of a control group treated with conventional titanium or standard tantalum implants further limits the ability to compare outcomes systematically. Second, the follow-up period averaged only 15.33 months, which is insufficient to evaluate the long-term durability of the implants, including the risks of late-onset loosening, wear, or prosthesis failure. Furthermore, due to the small sample size, the study was underpowered to detect subtle differences, and only basic statistical methods could be employed. Future studies should include larger patient cohorts and longer follow-up durations to validate the current results and assess the long-term effectiveness and safety of this personalized 3D-printed porous tantalum approach (Qian et al., 2022). This mainly includes further validating the effect of 3D-printed implants at different stages of rehabilitation and comparing the differences with traditional methods. In the field of basic research of Ta particles, our previous study has explored the deposition state of Ta particles after porous Ta implantation; investigated the regulatory effect of Ta particles on bone metabolism in human bone tissue; analyzed the effect of Ta particles on Mφ polarization, and it was verified that Ta particles affected the osteogenic ability of bone marrow stromal cells (BMSCs) by regulating the polarization of Mφ to M2 and the exosome secreted by M2 Mφ, which has provided insights for potential bone repair applications (Yang et al., 2024). However, our research on Ta is still not comprehensive, and related cell co-culture experiments are still ongoing, and more comprehensive results will be introduced to confirm the excellent biosafety and histocompatibility of Ta particles.

Future studies should focus on expanding the patient cohort to include a more diverse population and longer follow-up periods to assess the durability of the implants over time. Besides, exploring the cost-effectiveness of personalized 3D-printed implants compared with traditional methods could provide valuable insights for clinical decision-making (He et al., 2024). Research into further optimizing the design parameters for different types of bone defects and patient anatomies could also enhance the applicability of this technique.

## 5 Conclusion

This study indicates that personalized 3D-printed porous tantalum buttress is an effective method to address complex acetabular reconstruction in THA, especially for those with extensive acetabular bone defects. Advantages such as superior fit, enhanced stability, and minimized risk of complications highlight the potential for broader clinical applications and further research.

## Data Availability

The original contributions presented in the study are included in the article/supplementary material, further inquiries can be directed to the corresponding authors.
